# Neuromodulation of Prefrontal Cortex in Non-Human Primates by Dopaminergic Receptors during Rule-Guided Flexible Behavior and Cognitive Control

**DOI:** 10.3389/fncir.2017.00091

**Published:** 2017-12-05

**Authors:** Susheel Vijayraghavan, Alex J. Major, Stefan Everling

**Affiliations:** ^1^Robarts Research Institute, University of Western Ontario, London, ON, Canada; ^2^Department of Physiology and Pharmacology, University of Western Ontario, London, ON, Canada; ^3^Graduate Program in Neuroscience, University of Western Ontario, London, ON, Canada

**Keywords:** dopamine receptors, prefrontal cortex, working memory, rules, antisaccades, neuropharmacology, cognitive control, nonhuman primates

## Abstract

The prefrontal cortex (PFC) is indispensable for several higher-order cognitive and executive capacities of primates, including representation of salient stimuli in working memory (WM), maintenance of cognitive task set, inhibition of inappropriate responses and rule-guided flexible behavior. PFC networks are subject to robust neuromodulation from ascending catecholaminergic systems. Disruption of these systems in PFC has been implicated in cognitive deficits associated with several neuropsychiatric disorders. Over the past four decades, a considerable body of work has examined the influence of dopamine on macaque PFC activity representing spatial WM. There has also been burgeoning interest in neuromodulation of PFC circuits involved in other cognitive functions of PFC, including representation of rules to guide flexible behavior. Here, we review recent neuropharmacological investigations conducted in our laboratory and others of the role of PFC dopamine receptors in regulating rule-guided behavior in non-human primates. Employing iontophoresis, we examined the effects of local manipulation of dopaminergic subtypes on neuronal activity during performance of rule-guided pro- and antisaccades, an experimental paradigm sensitive to PFC integrity, wherein deficits in performance are reliably observed in many neuropsychiatric disorders. We found dissociable effects of dopamine receptors on neuronal activity for rule representation and oculomotor responses and discuss these findings in the context of prior studies that have examined the role of dopamine in spatial delayed response tasks, attention, target selection, abstract rules, visuomotor learning and reward. The findings we describe here highlight the common features, as well as heterogeneity and context dependence of dopaminergic neuromodulation in regulating the efficacy of cognitive functions of PFC in health and disease.

## Introduction

The prefrontal cortex (PFC) is a critical node in brain networks involved in complex cognitive functions and has undergone a great evolutionary expansion in primates (Wise, 2008). PFC dysfunction is implicated in cognitive deficits in a spectrum of neuropsychiatric disorders (Arnsten et al., 2012). Neurons in the PFC have been shown to possess persistent post-sensory activity in delayed response tasks, a feature which has been variously associated with short-term retrospective representation of salient stimuli or working memory (WM), spatial attention and representation of abstract rules (Fuster and Alexander, 1971; Goldman-Rakic, 1995; Wallis et al., 2001; Lebedev et al., 2004). In their pioneering study, Fuster and Alexander (1971) discovered neurons in PFC that had persistent activity in a short-term memory task. Subsequently, Goldman-Rakic and colleagues found that activity in the periprincipal region of PFC represented a briefly presented stimulus at contralateral spatial locations for several seconds (Funahashi et al., 1989), which they posited was the cellular basis of WM. Further investigations by various laboratories have found that PFC neuronal activity can, on a trial-to-trial basis, encode the relevant rule that is to be employed to execute a motor response (Wallis and Miller, 2003; Everling and DeSouza, 2005; Bongard and Nieder, 2010). In addition, periprincipal PFC neurons also show oculomotor-related activity during or after saccades towards a spatial goal (Funahashi et al., 1991). This activity has been shown in the neighboring frontal eye field (FEF) to encode a corollary discharge: a feedback signal from motor areas that informs cortical areas about a self-generated movement (Sommer and Wurtz, 2008).

The PFC is subject to substantial neuromodulation from all major ascending modulatory systems (Arnsten et al., 2012). The influence of dopamine on PFC function has been particularly well studied. Brozoski and Goldman-Rakic discovered the importance of dopaminergic innervation of PFC in a seminal study where they showed that dopaminergic deafferentation in PFC produced profound deficits in spatial WM, which were comparable to the effects of lesioning PFC itself (Brozoski et al., 1979). Since then, much progress has been made in understanding the mechanisms by which dopamine acts on various receptor subtypes to influence PFC neurons engaged in spatial WM (Sawaguchi and Goldman-Rakic, 1991; Williams and Goldman-Rakic, 1995; Vijayraghavan et al., 2007; Gamo et al., 2015). Most of our understanding of the physiological consequences of PFC neuromodulation has been accrued from microiontophoretic, local and systemic injection studies in the PFC of monkeys performing the oculomotor delayed response task. Recently, several groups have begun to explore the role of dopamine in other PFC-dependent functions, including top-down attention (Noudoost and Moore, 2011a; Soltani et al., 2013), visuomotor associative learning (Puig and Miller, 2012) and WM for abstract rules in numerical cognition and controlled oculomotor behavior (Jacob et al., 2013; Ott et al., 2014; Major et al., 2015; Vijayraghavan et al., 2016).

In this review article, we discuss recent investigations in our laboratory of the effects of dopaminergic neuromodulation of the PFC during the performance of rule-guided pro- and antisaccades (Vijayraghavan et al., 2016), and place them in the context of recent studies of neuropharmacology of prefrontal functions related to abstract rules, visual feature WM and spatial attention (also reviewed in Robbins and Arnsten, 2009; Arnsten et al., 2010, 2012, 2015; Noudoost and Moore, 2011b; Clark and Noudoost, 2014; Puig et al., 2015). We will focus on research conducted in nonhuman primates, with exceptions when relevant and pertinent data is not available from nonhuman primate models.

### Dopamine Modulation of Prefrontal Neurophysiology during Spatial Delayed Response Tasks

Interest in dopaminergic modulation of cognition had its incipience in the clinical observation in the 1970s that available neuroleptic medications targeted dopamine release, its receptors and downstream actions (Seeman and Lee, 1975; Carlsson, 1977), and the discovery of the dopaminergic innervation of rat medial PFC (Thierry et al., 1973). Dopaminergic innervation of the cortical mantle in PFC shows laminar heterogeneity (Lewis et al., 1988) with considerable variation during postnatal development (Lewis and Harris, 1991). In addition to extrasynaptic dopamine release (volume transmission), dopaminergic axons form synaptic specializations apposed to pyramidal dendritic spines in PFC (Smiley and Goldman-Rakic, 1993), and also specifically target parvalbumin-expressing interneurons (Sesack et al., 1998). Much of what we currently know about the anatomical profile and physiological role of dopamine in PFC function came from the early work of Goldman-Rakic and colleagues (Brown et al., 1979; Brozoski et al., 1979; Sawaguchi and Goldman-Rakic, 1991; Goldman-Rakic et al., 1992; Williams and Goldman-Rakic, 1993). Two families of dopamine receptors mediate dopamine’s actions, the G_s_-coupled D1 receptor (D1R) family comprising of D1 and D5 receptors and the G_i_-coupled D2 receptor (D2R) family comprised of D2, D3 and D4 receptors (Missale et al., 1998). D1Rs are expressed in supra- and infragranular PFC layers and are perisynaptically localized on dendritic spines of pyramids receiving asymmetric (glutamatergic) synapses (Smiley et al., 1994), while D5Rs are found on dendritic stems in close apposition to subsurface reticular specializations expressing IP3 receptors (Paspalas and Goldman-Rakic, 2004). D1Rs are also expressed presynaptically in glutamatergic axons targeting other pyramids but notably absent in projections targeting interneurons (Paspalas and Goldman-Rakic, 2005). D2Rs are more sparsely expressed in PFC, with labeling enriched in layer V (Lidow et al., 1998), and are expressed both presynaptically, on glutamatergic terminals, and postsynaptically in higher-order dendrites (Paspalas et al., 2006), thus in a position to regulate the physiology of subcortical outputs of the PFC. D2Rs are also classically identified as autoreceptors on dopaminergic afferents, and may subserve this function in PFC, though the extent of D2R expression on tegmental dopaminergic projections in PFC appears circumscribed compared with nigral afferents (Arnsten et al., 2009).

**Figure 1:**
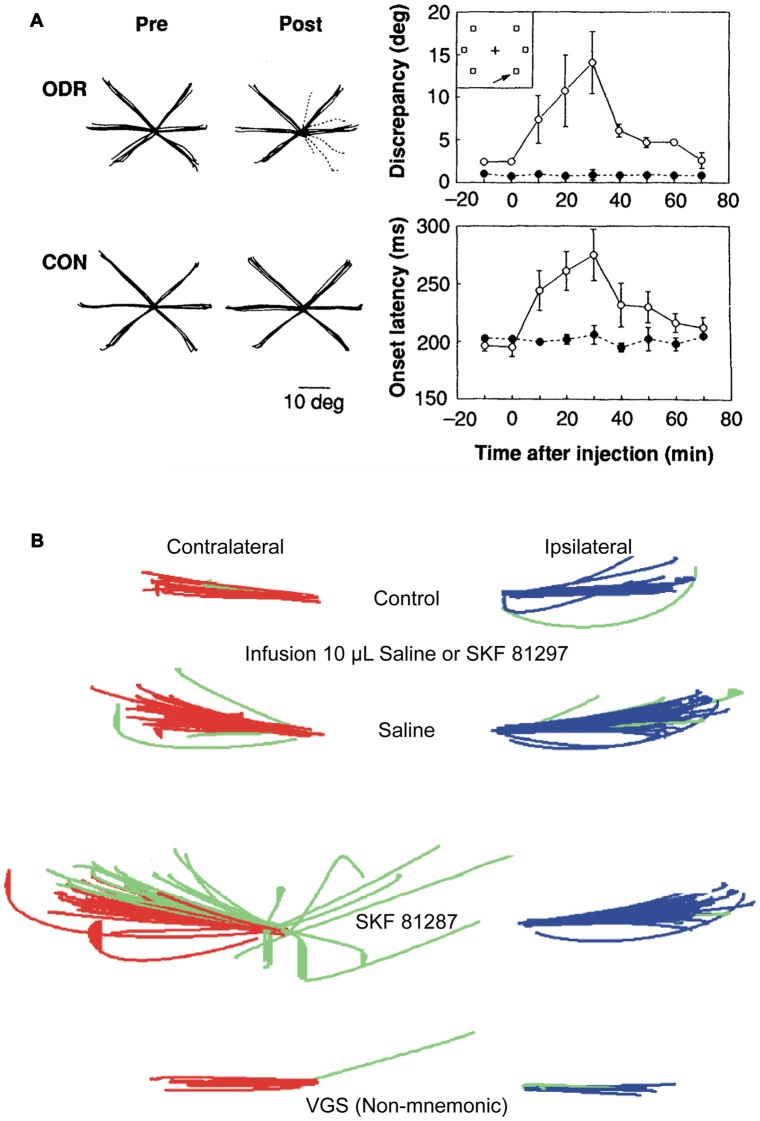
Effects of dopamine D1 receptor (D1R) blockade and stimulation in caudal dorsolateral prefrontal cortex (PFC) on oculomotor delayed response. **(A)** Local injections of D1R antagonist, SCH23390, induce deficits in contralateral memory-guided saccades, but not in visually guided saccades (Left panel; individual solid black lines are traces of correct saccades; dashed lines are erroneous saccades. Left most subpanels are control; right most sub panels are after drug injection. Top panels are during memory-guided saccade performance. Bottom panels are during control visually guided saccade performance with zero delay). Right panels show time course of drug-induced changes in angular dispersion of contralateral memory-guided saccades (top subpanel) and saccade reaction times (bottom subpanel). Panels adapted with permission from Sawaguchi and Goldman-Rakic (1991). **(B)** Local injections of D1R agonist, SKF81297, induce deficits in contralateral memory-guided saccades. Red traces show correctly executed saccades, green traces show error saccades (top panel, control; second panel, injection of 10 μl saline; third panel, infusion of 10 μl SKF81297; bottom panel, visually-guided saccades after SKF81297 injection). D1R stimulation, but not saline, induced increases in spatial dispersion of memory-guided saccades, but not visually-guided saccades to the contralateral target. Adapted from Gamo et al. (2015).

The first insights on how dopamine receptors modulate the physiology of macaque PFC neurons and their behavior came from the work of Sawaguchi et al. (1986), who found that iontophoretically applied dopamine mostly excited, but also inhibited PFC activity, primarily during motor responses in visual reaction time task. Dopamine also mostly excited, but occasionally inhibited the activity of PFC neurons during a delayed response task (Sawaguchi et al., 1990a), and increased the signal-noise of neurons with persistent activity during the delay epoch, and of neuronal activity in the GO-phase. In a subsequent study, Sawaguchi et al. (1990b) found that effects of dopamine iontophoresis on PFC neurons were reversed by D1R-preferring antagonist fluphenazine, and not by D2R-preferring antagonist sulpiride, suggesting that the primary effects of dopamine on PFC were mediated by D1Rs. We would like to note that, henceforth, when referring to pharmacological studies, we refer to the D1R family, since D1R family agonists and antagonists do not appear to distinguish between D1 and D5 receptors (Missale et al., 1998). Iontophoresis is the charge-induced ejection of minute amounts of drugs that affects a very localized cortical milieu in the vicinity of a recorded neuron, and thus, is not expected to have large behavioral effects, unlike delivery by pressure ejection of larger volumes of drugs. Microinfusions of larger volumes of specific D1R and D2R antagonists in PFC of rhesus monkeys engaged in oculomotor delayed response revealed that D1R blockade induced a “mnemonic scotoma”, a delay-dependent spatially restricted deficit in performance of contralateral memory-guided saccades (Figure 1A), while D2R antagonism had no effect on task performance (Sawaguchi and Goldman-Rakic, 1991, 1994). It is noteworthy though, that since monkeys under normal conditions perform this task with high accuracy, ceiling effects would preclude an assessment of potential performance enhancement with D2Rs. While local injections of pharmacological agents provide valuable information about neuromodulatory influences on the physiology of PFC during WM tasks, a caveat to this approach is that behavioral effects could be a consequence of imbalances in PFC networks, the coordinated activity of which underlies representations in WM (Salazar et al., 2012). However, systemic injections offer an alternative approach to study the behavioral consequences of neuromodulation. Systemic injection of D1R agonists in young and aged monkeys (who have lower endogenous dopaminergic tone) and intracortical microinfusions in rodents have delineated an “inverted-U” dose effect of D1R stimulation on delayed response performance. Low doses augment performance and higher doses cause deterioration (Arnsten et al., 1994; Zahrt et al., 1997). Microinjections of larger volumes of D1R agonist into PFC resulted in a delay-dependent contralateral deficit (Figure 1B) in memory-guided saccades (Gamo et al., 2015), thus establishing with Sawaguchi’s experiments described previously (Sawaguchi et al., 1990b), that too much or too little D1R stimulation is detrimental to spatial WM. Again, since monkeys perform this task natively with high accuracy, it was not possible to establish if local injections of low doses of D1R could improve performance. Paradoxically, systemic injections of a D2R agonist at low doses impaired spatial WM and at higher doses improved performance, but also induced motor dyskinesia and hallucinatory behavior (Arnsten et al., 1995). However, these systemic effects cannot be ascribed solely to PFC dysregulation, and could have a subcortical component.

Subsequent microiontophoretic studies delineated the physiological basis of this inverted-U response. Application of a highly selective D1R antagonist at low doses on PFC neurons enhanced their memory fields, while higher doses suppressed neuronal activity and eliminated memory period activity (Williams and Goldman-Rakic, 1995). Similarly, stimulation of D1Rs using a series of selective agonists revealed that the predominant effect of increasing D1R stimulation was inhibitory, with low doses enhancing spatial tuning by suppressing PFC memory activity for nonpreferred spatial targets, with relative sparing of preferred target activity, while higher doses completely suppressed the activity of PFC neurons (Vijayraghavan et al., 2007). Furthermore, that study found that application of a cyclic adenosine monophosphate (cAMP) signaling blocker reversed the suppression of PFC neurons induced by D1R stimulation. Thus, D1R stimulation elevated cAMP levels leading to neuronal suppression. Subsequently, blockade of the h-current, mediated by hyperpolarization-activated cation (HCN) channels was found to reverse cAMP-mediated neuronal suppression (Wang et al., 2007). Wang et al. (2007) hypothesized that cAMP production due to downstream G protein-coupled receptor signaling shifted the activation curves of HCN channels on dendritic spines to increase their open probability. In their open state, spine HCN channels contributed to shunting glutamatergic synaptic input, thereby leading to neuronal suppression. The Arnsten group has subsequently shown that D1Rs co-localize on dendritic spines with HCN channels and blockade of the HCN current could reverse D1R physiological suppression of PFC neurons (Gamo et al., 2015). In contrast to these physiological effects of D1Rs during spatial WM, relatively little is known about the physiological role of D2Rs. One clue emerged when iontophoresis of a D2R blocker and stimulator selectively disrupted and augmented, respectively, the activity of PFC neurons that discharge during the saccadic response epoch of memory-guided saccades, while sparing the persistent activity of “delay” neurons firing in the memory period prior to the response (Wang et al., 2004). This D2R-sensitive component was hypothesized to be a corollary discharge from superior colliculus, relayed through mediodorsal thalamus to PFC (ibid.).

Thus, studies interrogating oculomotor delayed responses in monkeys have established the profile of selectivity of dopamine modulation of PFC circuits. D1Rs, localized in infragranular and supragranular layers, are poised to regulate local and cortico-cortical PFC circuits that sustain cognitive representations with overall suppressive effects on neuronal activity. D2Rs regulate infragranular neurons projecting subcortically, and have a specific physiological effect of augmenting activity of neurons temporally linked to motor responses, while sparing persistent delay activity.

The actions of dopamine receptors on PFC neuronal physiology are legion, with actions on NMDA receptors, persistent sodium currents, presynaptic inhibition at glutamatergic synapses, and cell-type specific effects on inhibitory interneurons (Gorelova and Yang, 2000; Gao et al., 2001, 2003; Gorelova et al., 2002; Gao and Goldman-Rakic, 2003; Seamans and Yang, 2004; Trantham-Davidson et al., 2004). However, many of the excitatory mechanisms may already be fully engaged in awake, alert animals with basal endogenous catecholaminergic tone. A consensus is developing from *in vivo* studies in monkeys, that in awake behaving primates, D1Rs, and perhaps D2Rs, modulate neuronal excitability through their localized actions on dendritic spines co-expressing nonspecific cation and potassium channels, leading to a sculpting of excitatory input along the pyramidal dendritic arbor. This short-term non-structural form of modulatory plasticity has been termed dynamic network connectivity (reviewed in Arnsten et al., 2010, 2012, 2015).

Recently, there has been some contention about whether deficits found in oculomotor delayed responses after pharmacological manipulations and lesions in the studies described above indeed lead to a spatially localized mnemonic deficit, or are indicative of PFC involvement in more complex integration of information involving prior, current and future goals. Tsujimoto and Postle (2012) examined data from Wajima and Sawaguchi (2004), who injected GABA-A receptor antagonist bicuculline methiodide in macaque dorsolateral PFC while subjects performed oculomotor delayed response. While this is not an inactivation of PFC, it would be expected based on previous microiontophoretic and injection studies in delayed response tasks (Sawaguchi et al., 1989; Rao et al., 2000), that GABA-A blockade would cause aberrant activity in the PFC, in turn causing behavioral deficits. Wajima and Sawaguchi (2004) did, indeed find that monkeys made focal direction errors in oculomotor delayed response when the remembered stimulus was contralaterally presented. But the analysis performed by Tsujimoto and Postle (2012) revealed that the location where the erroneous saccades were made was influenced by the stimulus location on the previous trial. Moreover, the animals made corrective saccades to the current remembered location after making the erroneous saccade. This would suggest that the deficit in performance was not due to extinguished memory of the spatial location, but involved proactive interference from previous trials, and that PFC’s role in this task was more related to distinguishing the current coal from past goals (Tsujimoto and Postle, 2012). This interpretation would have implications for the studies of dopamine modulation of monkey PFC during spatial delayed response tasks, because previous studies have looked at single trial behavioral and physiological signatures, but there is no information about how trial structure and previous trial effects are affected by pharmacological manipulations. More systematic analyses of the nature of the deficit induced by pharmacological manipulations of PFC is required. Microinjections of dopamine receptor agonists and antagonists, and muscimol infusions need to be revisited to determine if monkeys with drug-induced oculomotor delayed response deficits do indeed, show evidence of post-error corrective saccades that would imply intact and accessible spatial mnemonic representations in other cortical areas (or unaffected PFC), and whether the location of erroneous saccades shows evidence of inter-trial interference.

Further, it is necessary to examine the influence of dopaminergic modulation on post-response, and intertrial activity, a theme which we will elaborate on further in the following sections.

### Dopamine Modulation of PFC Involvement in Spatial Attention

**Figure 2:**
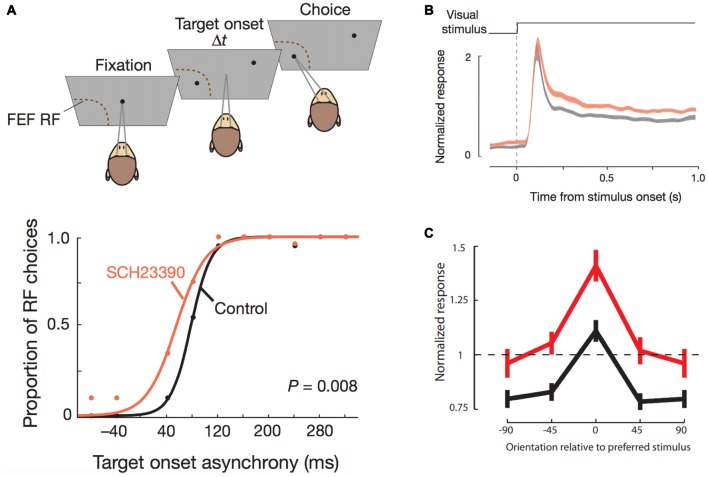
Effects of D1R blockade in frontal eye field (FEF) on saccade target selection and FEF modulation of V4 stimulus responsiveness. All panels adapted with permission from Noudoost and Moore (2011a). **(A)** Top panel shows the free-choice saccade task. Two stimuli are presented with varying temporal difference between their onsets. One stimulus is in the response field of the neuron recorded from the FEF area where pharmacological manipulations were performed. The monkey can freely choose between the two targets. Bottom panel shows the effects of D1R blockade on saccade choice. Traces (black, control; orange, drug) show the proportion of saccade choices towards the FEF response field for different temporal differences in onset of stimuli. D1R blockade increased the proportion of made towards the response field of the FEF area that was manipulated, thereby increasing contralateral saccade target selection. **(B)** Normalized stimulus-induced activity of V4 neurons with the same response field as the pharmacologically manipulated FEF area (black, control; orange, drug) increased after D1R blockade in FEF. **(C)** Orientation tuning of V4 neurons (black, control; red, drug) was augmented after FEF D1R blockade.

More recently, a spate of experiments in monkeys examining the role of dopamine receptors in other aspects of PFC functions have yielded novel insights, both corroborating and challenging theories that have emerged from the studies described above with spatial delayed response tasks. Noudoost and Moore (2011a,b) examined the effects of local D1R blockade and D2R stimulation using pharmacological microinjections in the FEF on saccade target selection in a free-choice saccade task. They simultaneously recorded activity from V4 neurons that had visual receptive fields matched to the receptive field of the FEF area in which dopamine receptors were manipulated. The FEF is an identified source of top-down attentional modulation signals to primary sensory cortices (Moore and Armstrong, 2003) and the authors, therefore, hypothesized that neuromodulation of FEF would likewise, have an impact similar to attentional modulation on receptive fields of V4 neurons. Intriguingly, they found that D1R blockade *and* D2R stimulation increased the selection of saccadic targets contralateral to the injection field, thus establishing opponent effects of the receptors in FEF on target selection (Figure 2A). Furthermore, notwithstanding the comparable effects on target selection, *only* D1R, and not D2R manipulation in FEF, affected the receptive fields of corresponding V4 neurons (Figure 2B). D1R blockade in FEF enhanced the orientation selectivity of V4 neurons (Figure 2C), while increasing activity and reducing trial-to-trial discharge variability. This is consistent with the anatomical localization of the D1R and D2R receptors in neighboring PFC (see above), wherein only D1Rs are expressed in layer II/III neurons with cortico-cortical connectivity that would include FEF-V4 projections (Schwartz and Goldman-Rakic, 1984). In a follow-up study (Soltani et al., 2013), the authors also found another interesting dissociation between the two receptors: D1R blockade of FEF decreased perseverative responding (repeat choices of the same location), while D2R stimulation strongly increased this tendency. It is worth bearing in mind that these dissociated effects on repetitiveness are over and above the target selection bias introduced by the pharmacological interventions. Interestingly, the authors reported that there were no effects on the metrics of saccades. The authors proposed a model whereby dopamine, acting through D1Rs in supragranular FEF with long-range cortico-cortical projections, can set the dynamic range of FEF-mediated attentional gain modulation and top-down control of visual cortical areas. In this model, saccade target selection enhancement observed upon D1R blockade and D2R stimulation is a consequence of their actions on D1Rs and D2Rs on infragranular neurons with subcortical projections. Since D2R stimulation did not affect V4 activity, the behavioral effects of FEF D2R stimulation were independent of the attention-like effects on V4.

Moreover, it was recently shown that antidromically identified macaque FEF neurons that project to V4 almost exclusively possessed spatial, delay period persistent activity for visual memoranda presented in their receptive fields (Merrikhi et al., 2017). These neurons with delay activity also notably lacked any saccadic activity. Intriguingly, this WM-like activity transmitted to V4 by FEF efferents did not result in changes in the discharge rates or activity of V4 neurons, but when the receptive fields of these V4 neurons were probed in the memory period at locations near the memorandum maintained in spatial WM, the authors found that the receptive fields of V4 neurons shifted towards the remembered location and the gain of the visual responses was enhanced. Similarly, the authors of that study also found that in another visual area, the middle temporal area, the firing rates of neurons did not change in the memory period, but the variability in trial to trial activity was reduced in the memory period.

These effects in primary visual areas are similar to the effects of D1R antagonist infusion in FEF on primary visual cortical activity. Since, the FEF neurons that directly project to primary visual cortical areas appear to mainly carry persistent spatially tuned delay activity, this implies that D1R blockade of these FEF projections cause the attention augmenting effects previously documented (Noudoost and Moore, 2011a) in primary visual cortex, including enhancement of visual receptive fields.

Thus, as described in the previous section, D1R modulation of local cortico-cortical recurrent networks in layer II/III of PFC showing spatial WM activity leads to deterioration in spatial delayed response performance, while D1R modulation of FEF long-range cortico-cortical circuits would account for attention enhancing effects in primary visual areas. There are a few points of interest to bear in mind when comparing these results in FEF with the previously described effects of similar microinjections in periprincipal PFC on memory-guided saccades. First, while D1R blockade in FEF increased saccade target selection and enhanced top-down attention-like effects in V4, almost certainly due to effects on corticocortical projections of FEF neurons with spatial WM activity, an identical manipulation (D1R blockade with the same drug) in neighboring periprincipal PFC, affecting neurons with identical delayed response activity, increased contralateral memory-guided saccade errors and reaction times (Sawaguchi and Goldman-Rakic, 1991, 1994; see “Dopamine Modulation of PFC Involvement in Spatial Attention” section), while visually guided saccades (no delay) were not affected. Thus, there appear to be differences between D1R actions in PFC on delayed saccades in a WM task and in FEF on saccade target selection and spatial attentional signals, even though D1R actions in both areas appear to be on neurons possessing delay period activity. It is additionally noteworthy that manipulations of dorsolateral PFC during the free-choice saccade task indicate that, PFC facilitates saccade target selection in a manner similar to FEF (Johnston et al., 2016). There has been vigorous contention regarding the relative importance of PFC, especially dorsolateral PFC (area 9/46 and 46) in retrospective short-term memory (Funahashi et al., 1993a; Funahashi, 2015), prospective motor preparation (Funahashi et al., 1993b; Takeda and Funahashi, 2002) and spatial attention/attentional selection (Rowe et al., 2000; Rowe and Passingham, 2001). Specifically, identifying the precise nature of information maintained in delay period activity in PFC has been a preoccupation in the field. Wise and colleagues, in an elegant experiment designed to dissociate between stimulus WM and attention, found that macaque PFC neurons encode the location of an attentional stimulus far more than the location of a stimulus held in WM (Lebedev et al., 2004). The dissociation described above between D1R manipulations in FEF on attentional effects and in PFC on spatial delayed response, then begs the question: if delay activity in dorsolateral PFC is more appositely described as a signature of maintenance of attention to a peripheral location which is utilized to select the saccade response, then why are there differences in the direction of behavioral effects between these studies employing the same D1R antagonist in dorsolateral PFC and in FEF, which are highly interconnected neighboring areas with apparently similar roles in saccade target selection?

Another, related point of interest concerns the physiological actions of dopamine. Data gathered in macaques from a series of iontophoretic studies (Wang et al., 2004; Vijayraghavan et al., 2007) and microinjections (Puig and Miller, 2012, 2014), suggest that the overall effect of moderate D1R blockade is mildly excitatory (likewise, D1R stimulation suppresses population activity). Thus, it would be reasonable to assume that both FEF D1R blockade during free-choice saccades tested by Noudoost and Moore (2011a) and D1R blockade in dorsolateral PFC during memory-guided saccades in Sawaguchi and Goldman-Rakic (1991) increased activity in the respective areas. So, increased activity in FEF due to D1R blockade enhanced increased signatures of attention and saccades to response field, while the identical manipulation (D1R blockade) in PFC, with presumably similar effects on neuronal physiology, led to inaccuracies in delayed saccades.

A trivial explanation would be that these differences in behavioral consequences of D1R blockade reflect an areal difference in dopamine’s modulation of the “spotlight of attention”. Alternatively, there may be differences in dopaminergic modulation of  “immediate” vs. “delayed” selection of action targets: enhancement of response field stimulus selection could be positively modulated by D1R blockade when the stimulus is visible, whereas the introduction of a delay would cause the opposite effect, perhaps due to increases in variability of cortical representations in the intervening delay period leading to deterioration in attentional representation of the stimulus location. These divergent pharmacological effects appear to highlight the heterogeneity of D1R actions on different cortical areas. Future experiments employing similar microinjections in a task similar to Lebedev et al. (2004) may shed light on this interesting dissociation between dopamine effects on these neighboring and highly interconnected cortical areas. It is also necessary to perform D1R manipulations in FEF and DLPFC while testing both for spatial WM performance and attentional selection of response goals. An interesting possibility would be to study dopamine modulation in a task that incorporates both WM and response selection between competing alternatives, like the free-choice saccade task recently explored by Mochizuki and Funahashi (2016), where PFC activity was found to predict the eventual free choice of a saccadic goal.

### Actions of Dopamine on Associative Learning and Reward in PFC

Dopamine has a well-established role in reward signaling in the brain (Schultz et al., 1993a,b). In addition to a tonic mode of activity, dopaminergic neurons have phasic responses to rewards and reward-predicting stimuli (ibid.). Many addictive drugs of abuse have direct effects on dopamine levels or signaling (Wise, 1994). Phasic dopamine activations encode the uncertainty in reward relative the prediction of reward: the “reward prediction error” (Schultz et al., 1997). During learning, phasic activations of dopaminergic neurons that follow a reward, temporally shift, such that later in the course of learning, activations occur after onset of reward predicting stimuli, and cease to occur for the reward itself. Thus, in fully trained monkeys performing spatial delayed response, dopamine neurons fire during the presentation of the cue that will determine the subsequent rewarding action, but do not show sustained firing in the delay period of the task (Schultz et al., 1993a).

Ventrolateral PFC neurons are modulated by reward, and spatial selectivity of neurons therein is modulated by the reward contingency (Kennerley and Wallis, 2009). Reward magnitude affects neuronal responses in dorsolateral PFC during spatial delayed response performance, but reward modulation is only observed after the appearance of a spatial cue to be maintained in WM (Leon and Shadlen, 1999). When the cue that indicated the expected reward was presented prior to the spatial cue in the course of the trial, PFC activity reflected the expected reward only after onset of the spatial cue (ibid.). Thus, reward modulated the *gain* of spatial tuning in the memory period, a phenomenon reminiscent of attentional modulation. Interestingly, Leon and Shadlen (1999) found that the modulation in PFC persisted during the memory period, while cue-contingent reward modulation in FEF did not persist in the memory period. Work from the Hikosaka group showed that FEF representation of spatial cues was modulated by reward in an asymmetric reward task, where the same spatial cue was rewarded differently in blocks of trials (Ding and Hikosaka, 2006), and they further confirmed that such reward-cue position effects in FEF do not persist in the delay period of the task. Thus, there are subtle differences in reward modulation of PFC and FEF, which is interesting given the discussion above about the effects of D1R antagonists in dorsolateral PFC and FEF. Further, the effects of D1Rs and D2Rs on reward modulation of saccadic movements in the caudate nucleus are dissociable, with D1R antagonists attenuating reward modulation effects on saccade reaction times and D2R antagonists augmenting such modulation (Nakamura and Hikosaka, 2006). These findings are reminiscent of the effects of the receptors described above in FEF by the Moore group (Noudoost and Moore, 2011a).

Two studies examined the role of PFC D1Rs and D2Rs in associative learning (Puig and Miller, 2012, 2014; reviewed in Puig et al., 2015). In the task, the monkeys learned to associate novel visual objects with saccades made to visual cues to the left or right after a delay period. The authors found that local PFC blockade of either D1Rs or D2Rs impaired learning of new visuomotor associations, while having no effect on the performance of previously well-learned associations in monkeys. The monkeys performed a task where stimuli were associated with specific saccades. However, there were also subtle differences between the effects of the two antagonists. Microinjections of the D2R antagonist affected learning of new associations when injected in dorsolateral or ventrolateral PFC, while the D1R antagonist only had effects when injected in ventrolateral PFC. The authors reason that this was due to differences in the lipophilicity of the drugs affecting extent of diffusion of the drugs (Puig et al., 2015). However, the difference could also be due to involvement of D2Rs in regulating perisaccadic activity in dorsolateral PFC (Wang et al., 2004), whereby the blockade of either receptor can lead to deficits in forming novel stimulus-saccade associations, but may do so by affecting different aspects of the cognitive circuitry. In this scenario, perhaps D2R blockade in dorsolateral PFC affected feedback signaling about the rewarded or unrewarded saccade aiding saccade target selection, thus disrupting learning. In contrast, D1R may have affected the integration of the remembered stimulus into learned stimulus-response associations in ventrolateral PFC, a locus critical to such associations (Bussey et al., 2001), possibly due to the preponderance of stimulus representation therein (Wilson et al., 1993).

Puig and Miller (2012, 2014) also recorded from multiple neighboring neurons (<2 mm from the injection site) to examine the activity of PFC neurons during drug infusion. They found that, like other iontophoretic studies described above (Vijayraghavan et al., 2007, 2016; Ott et al., 2014; Ott and Nieder, 2017), the D1R antagonist increased neuronal activity, and additionally, more so for the nonpreferred saccade direction. D2R blockade, in contrast, reduced neuronal activity, consistent with previous studies (Wang et al., 2004).

Thus, injections of antagonists of both dopamine receptor families disrupted novel visuomotor learning involving associating objects with saccades in particular directions, but did not affect familiar and well-learned associations. This is interesting when considering effects of injection of these antagonists in periprincipal PFC on oculomotor delayed response performance, as discussed in the previous section (Sawaguchi and Goldman-Rakic, 1991, 1994), wherein the D2R antagonist did not have appreciable effects on performance. If one were to think of an oculomotor delayed response as a well-learned association between the spatial position of a visual stimulus and a delayed motor response to (in this case) the same location, the experiments discussed above imply that the D1R antagonist affects this well-learned association between a spatial stimulus and a saccadic response, while sparing a well-learned association between foveally presented visual features and an arbitrary saccade to a location. Thus, in one situation, D1R blockade only disrupts acquisition of rewarded saccadic associations, while in the other case, it affects steady state performance in an overlearned saccadic task, in which both the sensory goal and response were spatial in nature. Alternatively, it could be that because oculomotor delayed response involves spatial WM, and feature-based well-learned visuomotor associations do not, pharmacological manipulation of PFC in the overlearned oculomotor delayed response task produces changes in performance. An interesting question is: what would happen with D1R antagonist injections in dorsolateral PFC in a task where spatial visuomotor associations are flexible? Will D1R antagonists only affect the learning of such associations, or also affect the performance of well-learned associations? Another question concerns the mechanisms of D1R actions in the context of learning acquisition and steady-state post-learning performance. It appears, as discussed before, that short-term plasticity phenomena which affect physiology of prefrontal neurons on the time-scale of a session may be responsible for changes in steady-state performance of well-learned tasks due to D1R manipulations. Could the overall physiological effects of D1R manipulations on PFC neurons that account for effects on steady-state spatial WM performance, also account for effects on within–session learning of novel visuomotor associations? Experiments involving manipulations analogous to those performed by Nakamura and Hikosaka (2006), with pharmacological manipulation of PFC during tasks where asymmetric rewards are paired with saccades to specific locations may be useful to ascertain if dopamine manipulations in PFC have similar effects to those in the striatum. Finally, the effects of dopaminergic pharmacology on post-response activity and in the intertrial intervals of tasks needs to be examined in further detail. Particularly, understanding the effects of D1R and D2R manipulations on postsaccadic activity in contexts where the saccades to different locations are asymmetrically rewarded will be helpful in resolving the role of PFC dopamine in reinforcement learning, steady state performance and response-outcome representations (Tsujimoto and Sawaguchi, 2004).

### The Role of PFC Dopamine in Maintenance of Cognitive Sets and Abstract Rules

Another critical role that has been associated with PFC is in the maintenance of cognitive sets: learning, maintenance and application of sets or rules used to guide flexible responses to environmental stimuli (Wallis and Miller, 2003; Everling and DeSouza, 2005; Chudasama and Robbins, 2006; Robbins and Arnsten, 2009). The PFC is also crucial to the integrity of set shifting, as is evidenced by disrupted performance in the Wisconsin Card Sort Task in subjects with PFC lesions (Owen et al., 1991). Buckley et al. (2009) examined the effects of aspiration lesions of different PFC regions in macaques on the performance of a simplified paradigm that recapitulates the principle features of the Wisconsin Card Sort Task. They found lesions of the anterior cingulate cortex, orbitofrontal cortex and periprincipal cortex all affected performance in the task. However, lesions of the periprincipal cortex caused deficits in performance when the intertrial interval was increased, thereby delineating a role for this region in maintaining the relevant task rule in WM. Interestingly, lesions of the area dorsal to the principal sulcus did not result in any deficits in task performance. Cryogenic inactivation of periprincipal cortex has been found to cause deficits in an uncued version of the pro- and antisaccade task (Hussein et al., 2014), where, similar to the paradigm in Buckley et al. (2009), the relevant rule is not specified, but updated based on reward feedback. Hussein et al. (2014) also found that inactivation of cortex dorsal to the principal sulcus does not affect performance in the uncued task. Thus, PFC involvement in rule maintenance is specialized by cortical region and specific to task attributes. In an intriguing study, catecholamine deafferentation with 6-hydroxy dopamine in marmoset PFC had complex effects on acquisition and maintenance of attentional sets and set shifting (Roberts et al., 1994). Roberts et al. (1994) found that extradimensional set shifting performance was enhanced by catecholamine deafferentation of PFC, which however, degraded spatial WM performance, but had no effect on reversal learning. However, in a subsequent study, the authors found that catecholamine deafferentation of PFC impaired the acquisition and distractor-resistant maintenance of attentional sets (Crofts et al., 2001). PFC excitotoxic lesions, but not dopamine deafferentation of PFC, disrupted performance in a self-ordered sequencing task (Collins et al., 1998), notwithstanding disruption of spatial WM in the deafferented monkeys. Thus, dopamine modulation of PFC regulation appears to be selective to the task context and whether parametric (e.g., spatial WM or selective attention) or categorical (e.g., cognitive set, task rule) stimuli are being manipulated in the task (Arnsten, 2011).

**Figure 3:**
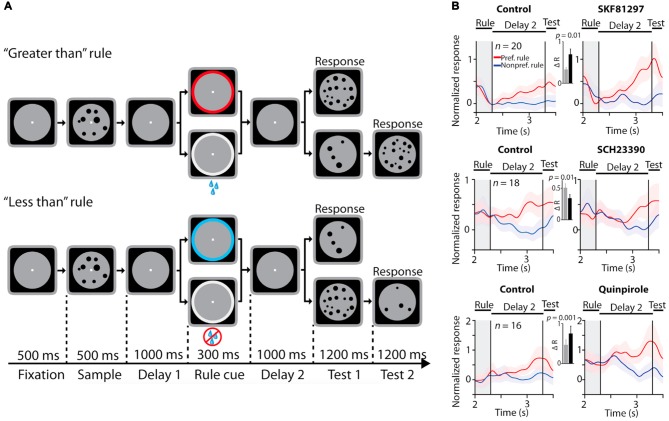
Effects of dopamine receptor manipulations on PFC activity encoding rules in a numerosity judgment task. All panels adapted with permission from Ott et al. (2014). **(A)** Rule-guided numerosity judgment task used in Ott et al. (2014). Macaques had to use a rule (“greater than” or “less than”) cued by a colored dot or drops of juice to determine if a test numerosity panel displayed after a delay had more or less dots than a previously presented sample numerosity panel. The animals responded by holding or releasing a lever, based on their trial numerosity judgment. **(B)** Effects of D1R stimulation (top panel), D1R blockade (middle panel) and D2 receptor (D2R) stimulation (bottom panel) on normalized activity of PFC neurons showing differential responses to the trial rule. D1R stimulation and D2R stimulation augmented selectivity in rule representation, while D1R blockade suppressed population rule selectivity.

PFC neurons maintain information about the cognitive set and the rule to be employed in a behavioral trial (Wallis et al., 2001; Everling and DeSouza, 2005). This activity is also maintained in WM. It has since become apparent that PFC neurons encode and maintain task set information in a diversity of task contexts (Duncan, 2001). Furthermore, PFC neurons directly convey such information to other cortical areas and modulate the activity of subcortical regions that are involved in generating motor commands (Johnston and Everling, 2009). Recently, a series of studies from different groups have examined the role of dopamine receptors in physiological modulation of such task-rule activity in PFC (Ott et al., 2014; Vijayraghavan et al., 2016; Ott and Nieder, 2017). Ott et al. (2014) iontophoretically applied a D1R agonist, a D1R antagonist and a D2R agonist on rhesus monkey PFC neurons engaged in a rule-guided numerical comparison task (Figure 3A), where the rule specified whether to assess the numerosity of a sample comprised of an array of dots as “less than” or “greater than” another array of dots presented after a delay. The task design involved the maintenance of the rule in WM as well. They examined the effects of these compounds on PFC neurons that showed differential activity for “less than” vs. “greater than” rule selectivity. In their paradigm, they tested the D1R agonist at doses comparable to doses that showed the peak “inverted-U” effect in previous studies with spatial delayed response (see above; Wang et al., 2004; Vijayraghavan et al., 2007). They found that the D1R agonist suppressed, and both the D1R antagonist and the D2R agonist increased baseline activity, consistent with previous studies. However, they found that D1R stimulation increased activity for the preferred rule, thereby augmenting the selectivity for the rule during the delay period, while the D1R antagonist application had the opposite effect (Figure 3B). These findings are in contrast to low dose D1R agonist application during spatial delayed response (Vijayraghavan et al., 2007), where it was found that D1R stimulation had suppressive effects for both preferred and nonpreferred spatial directions, but suppressed activity more so for nonpreferred directions. Further, the authors found that D2R stimulation also increased selectivity for rule representations in WM, but this was effected by comparative suppression of responses for nonpreferred rule activity (Figure 3B, lower). It is noteworthy that these comparisons were made on normalized responses, i.e., the baseline activity of the neurons was augmented with D2R application. These physiological effects are consistent with previous reports of application of D2R agonist quinpirole on PFC neurons (Wang et al., 2004), which found that D2R stimulation has an overall excitatory effect. However, Wang et al. (2004) found that D2R selectively modulated activity in neurons which had activations in the perisaccadic epoch while neurons with delay period activity were impervious to stimulation or blockade of D2R. The enhancement of rule WM signals by D2R stimulation found in Ott et al. (2014) delineates differences between its effects on rule and spatial WM. In addition to rule coding, the authors found that selectivity for the numerosity in the sample stimulus, or “numerosity coding”, was enhanced by D1R stimulation, but unaffected by D1R blockade or D2R stimulation. In a subsequent set of studies (Jacob et al., 2016; Ott and Nieder, 2017), the Nieder group further examined effects of dopamine receptor stimulation on PFC activity related to stimulus maintenance in WM. Jacob et al. (2016) examined the influence of D1R modulation on maintenance of stimulus (dot array) numerosity selectivity under distractor load in putative pyramidal and interneurons (defined based on waveform shape) in PFC. They found that, in putative pyramidal neurons, D1R blockade enhanced restoration of target selectivity in neuronal activity after distractor interference, while D1R stimulation had the opposite effect, disrupting target selectivity after distractor interference. It is noteworthy that, based on signal detection theory analysis, they also found D1R stimulation decreased target selectivity before the distractor. In another study, Ott and Nieder (2017) examined the effects of D1R and D2R stimulation on selectivity for the target (dot array) in WM. They found that D2R stimulation increased numerosity target selectivity during the delay period. In contrast, D1R stimulation or blockade did not affect numerosity representation in WM. The latter finding is intriguing, because Jacob et al. (2016) found enhancement of numerosity sample selectivity with D1R blockade in the delay period prior to distractor onset (see red vs. black traces, Figures 4C,F of Jacob et al., 2016). Although the task paradigm was slightly different, with the appearance of a distractor after 1 s of delay, the initial delay epoch in both tasks were equivalent. It is possible that the apparent discrepancy is due to subtle changes in task demands imposed by the distractor.

**Figure 4:**
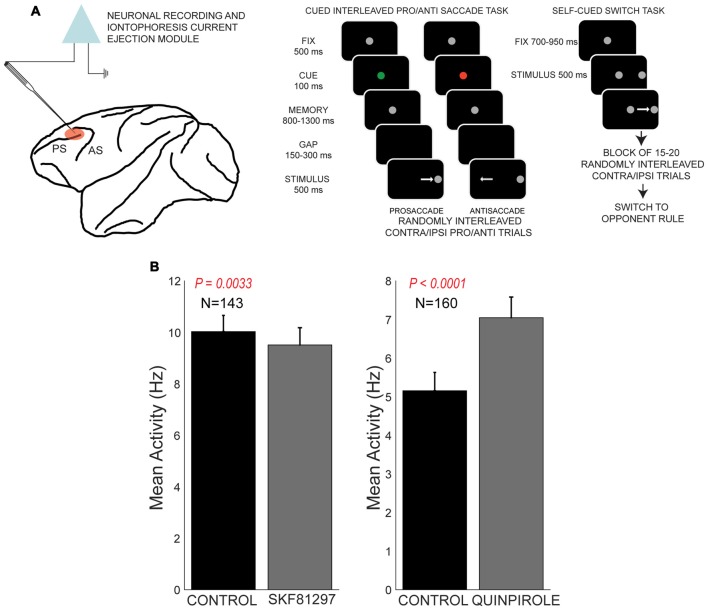
Effects of D1R agonist and D2R agonist on population activity of PFC neurons recorded during the performance of the rule-guided pro- and antisaccade task. All panels adapted with permission from Vijayraghavan et al. (2016). **(A)** Recording locus (left) and the pro- and antisaccade task are shown. Subjects maintained central fixation, and a briefly presented colored cue indicated the rule to employ in the trial. This information was maintained in working memory (WM). After a brief gap post-fixation point offset, a peripheral stimulus was presented and monkeys had to use the previously remembered rule to make a saccade towards or away from the stimulus. In another version of the task employed in Vijayraghavan et al. (2016), the rule was uncued and the subjects had to derive the trial rule based on reward feedback and maintain this rule was a block of trials. Blocks of prosaccade trials alternated with blocks of antisaccade trials and the subjects learned to switch response types based on feedback. **(B)** D1R stimulation (left panel) with iontophoresis of full D1R agonist SKF81297 suppressed the population activity of PFC neurons, while D2R stimulation with D2R agonist quinpirole (right panel) increased population activity.

Recently, our group examined the influence of dopamine receptor subtypes on rule representation in PFC (Vijayraghavan et al., 2016) in a task that required rule-contingent oculomotor responses in monkeys (Figure 4A). Monkeys were required to maintain a briefly displayed rule cue in WM and then utilize that rule to make a saccade towards a subsequently presented peripheral stimulus (prosaccade), or suppress that automatic response, and look away from the stimulus towards the opposite location. These so-called antisaccades are dependent on PFC integrity in macaques (Wegener et al., 2008; Koval et al., 2011; Johnston et al., 2016) and patients with lesions of frontal cortex (Guitton et al., 1985; Fukushima et al., 1994; Rivaud et al., 1994) also exhibit deficits in the antisaccade task. Muscimol inactivation of periprincipal PFC in monkeys also causes deficits in antisaccade performance (Condy et al., 2007). Moreover, patients with psychiatric conditions including schizophrenia and attention deficit hyperactivity disorder exhibit increased error rates in this task, and antisaccade task performance is a consistent correlate of the extent of cognitive symptoms in schizophrenia (Fukushima et al., 1988, 1994; Klein et al., 2003). The antisaccade task recruits the brain’s oculomotor network, including the superior colliculus (Everling et al., 1999), FEF (Everling and Munoz, 2000), lateral intraparietal area (Zhang and Barash, 2000) and dorsolateral PFC (Funahashi et al., 1993b; Everling and DeSouza, 2005). The dorsolateral PFC displays rule-dependent activity in this task (Everling and DeSouza, 2005) and sends task-related signals directly to the superior colliculus (Johnston and Everling, 2006).

**Figure 5:**
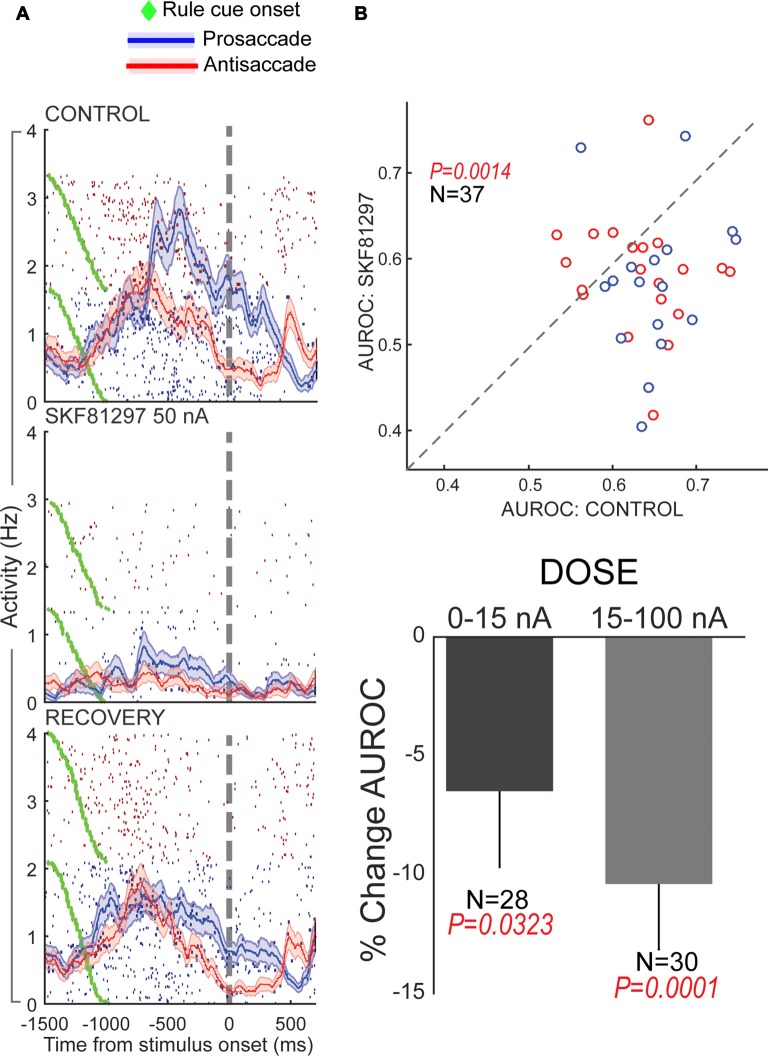
Effects of D1R stimulation on rule selectivity of PFC neurons. All panels adapted with permission from Vijayraghavan et al. (2016). **(A)** Iontophoretic stimulation of D1R (middle panel) suppressed the activity of a PFC neuron that had more prestimulus activity during prosaccade trials. Rule selectivity of the neuron was diminished, and recovered upon cessation of drug application (bottom panel). Blue traces and rasters: prosaccade trials; Red traces and rasters: antisaccade trials. **(B)** D1R stimulation decreased PFC population rule selectivity (top panel) assessed by signal detection theory. The area under the receiver operating characteristic curve (AUROC) for individual rule-selective neurons tested is plotted for control (abscissa) and during D1R stimulation (ordinate). Drug application shifted the AUROCs below the equality line (dashed line) indicating reduction in population rule selectivity. D1R stimulation at both low and high doses reduced population rule selectivity, with greater reduction for higher doses (bottom panel). Blue dots represent neurons with greater activity for the prosaccade rule and red dots, neurons with greater activity for the antisaccade rule.

Using iontophoresis, we examined the effects of D1R and D2R stimulation on the activity of dorsolateral PFC neurons engaged in this task. We tested two agonists, SKF81297 and quinpirole, and examined the overall effects and dose-dependence on PFC excitability and task representation. D1R stimulation had an overall suppressive effect on the population of PFC neurons tested, while D2R stimulation had an overall excitatory effect (Figure 4B), consistent with other studies discussed in this review article. Although we found individual neuronal counter-examples, there were consistent effects on population activity after these recepor manipulations. When we examined the selectivity for the trial rule in the delay period, higher doses of D1R stimulation disrupted overall rule selectivity of the population of “rule neurons” that distinguished between pro- and antisaccade task rules in their activity (Figure 5). We also examined D1R stimulation at lower dose ranges to examine if there were “inverted-U” effects on rule selectivity in PFC. Low dose D1R stimulation did not improve rule selectivity and to the contrary, also disrupted rule selectivity, but to a lesser extent than higher dose stimulation. Thus, it appeared D1R stimulation in this study did not have inverted-U effects on rule selectivity, in contrast to the effects found for spatial delayed response in Vijayraghavan et al. (2007) and rule-selectivity effects found in Ott et al. (2014). Further, D2R stimulation of individual PFC rule neurons showed both augmentation and deterioration of rule selectivity in the delay epoch. However, population analysis did not show appreciable consistent effects on rule selectivity.

Our results with dopamine receptor modulation of rule selectvity were different from those found in the study by Ott et al. (2014). One explanation may be different task demands, which may change the endogenous dopaminergic tone in PFC, thereby influencing the outcome of the pharmacological manipulations (Arnsten, 1998; Robbins and Arnsten, 2009; Arnsten et al., 2012). The numerical cognition task employed in Ott et al. (2014) is more complex than the rule-antisaccade paradigm in our study, as it involves two delays and the simultaneous maintenance of rule and stimulus in WM. Another reason for this discrepancy might be that the exact subzones of PFC where the two groups were recording might have been slightly different, although there appears to have been some overlap. Another point to bear in mind is that, while both tasks are based on rules to make contingent motor responses to stimuli, the task in Ott et al. (2014) presumably involved feature based stimulus processing and WM, and the task in Vijayraghavan et al. (2016) involved processing the spatial location of a visual stimulus, a modality in which the dorsolateral periprincipal PFC has a well established role, which we have discussed extensively in this review article. Furthermore, the motor responses involved in the two studies were also different. In Ott et al. (2014), the response was a manual lever release, whereas, in our paradigm, the response was a spatially directed saccade. Now, while it may be argued that the stimulus and motor modalities should not impinge on the maintenance of the rule, which is a cognitive operation, it is possible that neuronal activity in PFC for rules may manifest in a specific way depending on task context, possibly because the nature of inputs contributing to the task feature, in this case rules, may be very different in different contexts. Indeed, this is borne out in the dissociable effects of PFC inactivation on cued and uncued pro- and antisaccade tasks (Hussein et al., 2014), discussed previously. This, in turn, may affect the way in which neuromodulation shapes task representations in PFC. We would also like to emphasize that the oculomotor task we employ here has been shown to be dependent on PFC integrity (see above) and the connectivity, lesions, pharmacological manipulations and prior physiology that we have discussed in this review highlight the fact that the cortex surrounding caudal principal sulcus is intimately involved in processing of spatial information and goals. It is not known, however, whether the numerical comparison task employed by Ott et al. (2014) is causally dependent on activity in the periprincipal dorsolateral PFC, though the PFC lesion studies of Buckley et al. (2009) suggest a role for this cortex in WM maintenance of rules, regardless of an oculomotor context. As discussed previously in this review, the effects of dopamine on maintenance of attentional sets and rules seem to be highly sensitive to task context, and perhaps the dichotomies found between our results on dopamine receptor modulation of rule activity and those of Ott et al. (2014) may be another example of this heterogeneity. Future experiments involving larger microinjections of these agonists in PFC to measure effects on behavioral performance in the respective tasks may help resolve these divergent results regarding the dopamine agonists.

**Figure 6:**
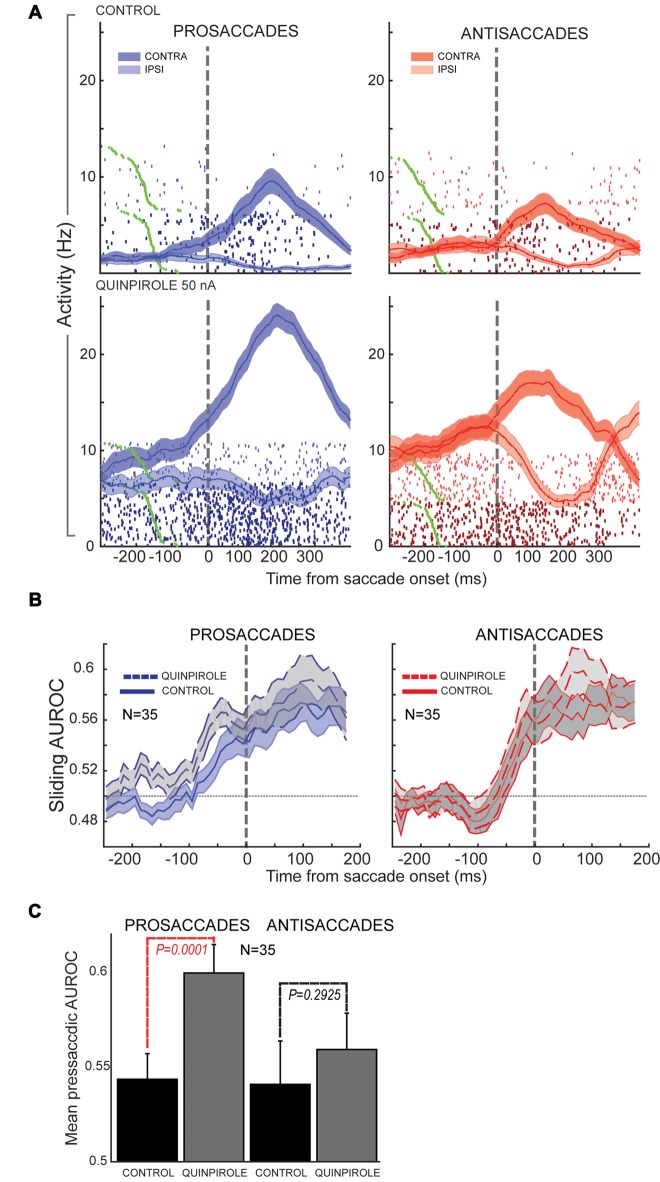
Effects of D2R stimulation on perisaccadic activity of PFC neurons during pro- and antisaccades. All panels adapted with permission from Vijayraghavan et al. (2016). **(A)** Trial rasters and mean spike density functions for a PFC neuron with elevated perisaccadic activity for contralateral saccades are shown. D2R stimulation increased the activity of the neuron, but had a greater effect on perisaccadic discharges during prosaccade trials (left; dark blue, contralateral prosaccade; light blue, ipsilateral prosaccade) than during antisaccade trials (right; dark red, contralateral antisaccade; light red, ipsilateral antisaccade). **(B)** Sliding population saccade selectivity analysis shown for 35 PFC neurons with contralateral saccade selectivity. D2R stimulation increased saccadic selectivity prior to saccade onset (dashed line) for prosaccades, but not antisaccades. Saccade selectivity was not appreciably affected after the saccade. **(C)** Population saccade selectivity in the presaccadic epoch was increased for prosaccades but not for antisaccades after D2R stimulation (control, black; gray, drug).

In our study (Vijayraghavan et al., 2016), we further examined the effects of D1R and D2R agonists on stimulus direction selectivity (selectivity for the peripheral stimulus that triggers the contingent saccade) and saccade direction selectivity. We found that D1R stimulation increased contralateral selectivity for the stimulus direction in the PFC neuronal population that possessed visual selectivity. D2R stimulation had no effect on this facet of the task. However, D2R stimulation augmented saccade direction selectivity of PFC neurons with perisaccadic activity, which was most prominent after the saccade. When we examined this effect further, looking at contralateral saccade direction selectivity on prosaccade trials and antisaccade trials separately (Figure 6, examining identical saccades in the two trial contingencies), we found that the D2R effect on saccade direction selectivity was more prominent for prosaccades than antisaccades (where it did not reach significance). Moreover, when evaluating the time course of selectivity changes, we found that D2R stimulation increased saccade direction selectivity on prosaccade trials more prominently in the presaccadic epoch, shifting the onset latency and peak of the perisaccadic activation. Thus, saccade selectivity enhancement by D2R stimulation was sensitive to the trial rule and time course of the trial.

A previous study found that D2Rs selectively modulated the activity of neurons that were only activated during the saccade response in the spatial delayed response task (Wang et al., 2004). D2R stimulation augmented the activity of these neurons, but not neurons that showed persistent activity in the delay period. Wang et al. (2004) hypothesized that D2Rs selectively gate neurons that receive corollary discharge feedback signals from the superior colliculus, relayed through mediodorsal thalamus (Sommer and Wurtz, 2002). The antisaccade task affords the opportunity to examine saccade-related activity for the same saccade in both a stimulus-driven context (prosaccade) and an internally-driven context (antisaccade). If perisaccadic activity in the PFC represents corollary discharge signals from the superior colliculus, which are then used to remap cortical retinotopic spaces based on the current eye position signal, we would expect that D2R modulation of such activity should be independent of whether it occurred for a prosaccade or antisaccade, which is contrary to what we observed (Vijayraghavan et al., 2016). Herein, we examine a few possible explanations. One possibility is that corollary discharge signals generated in the superior colliculus are themselves subject to modulation by the task set. It is known that preparatory activity is influenced by task set in the superior colliculus (Everling et al., 1999) and that stimulus- and saccade-related activity is reduced on antisaccade trials. This difference in saccade activity between the trial types could be relayed faithfully via mediodorsal thalamus to PFC, where D2R actions would differentially amplify this difference due to multiplicative scaling of gain. However, activity in the mediodorsal thalamus recorded in the antisaccade task shows that mediodorsal neurons do not systematically have smaller saccadic bursts on antisaccade trials than prosaccade trials (Kunimatsu and Tanaka, 2010), which makes the possibility described above less likely. There is also other evidence that postsaccadic activity in the PFC is not a corollary discharge signal from tectal saccade-generating circuitry. Funahashi et al. (1991) undertook a study of perisaccadic activity of monkey PFC neurons during delayed saccades, visually-guided saccades and spontaneous saccades with identical metrics made between trials. They found that PFC presaccadic and postsaccadic discharges are present only when the monkeys execute purposive saccades in the task, and not when identical spontaneous saccades were made. Thus, there is context-dependence to PFC perisaccadic activity. In contrast with this, in the FEF only presaccadic activity is similarly context-dependent, while postsaccadic discharges are always present, regardless of the context in which the saccade was executed (Bruce and Goldberg, 1985). Similary, neurons in the superior colliculus have discharges for saccades, regardless of whether the saccade was spontaneous or purposive (Sparks and Mays, 1980). Thus, corollary discharge signals from the superior colliculus should be context-independent and are relayed to the FEF (Sommer and Wurtz, 2002), where postsaccadic discharges are not context-dependent. However, since PFC postsaccadic discharges do not manifest in spontaneous saccades, they cannot be corollary discharge (Funahashi et al., 1991).

What then are the origins and functional role of perisaccadic activity in dorsolateral PFC, and thereby, what is the role of D2Rs in the neuromodulation of this activity?

One possibility is that, D2Rs modulate the presaccadic component of perisaccadic activity that is involved in saccade target selection. PFC neurons exhibit greater representation of the contralateral visual space in their stimulus-driven activity (Funahashi et al., 1990; Bullock et al., 2017). In this scenario, D2R modulation would have a greater effect on saccade selectivity for prosaccade trials, because it would strengthen feedforward microcolumnar connections between visually-driven neurons responding to the contralateral stimulus (or neurons representing the stimulus with post-sensory activity) in PFC and layer V neurons that are providing outputs to the brainstem. During antisaccade trials, since the visual stimulus is predominantly represented in the other hemisphere (Bullock et al., 2017), similar augmentation would not occur. This model would be congruent with the results of Noudoost and Moore (2011b) (see discussion above), showing that D2R stimulation augments saccade target selection. Interestingly, a recent study showed that PFC deactivation reduced saccades made to the contralateral hemifield in the same free-choice saccade task employed by Noudoost and colleages (Johnston et al., 2016). This delineates a role for periprincipal PFC in saccade target selection. Our hypothesis regarding D2R involvement in target selection in PFC, in addition to the FEF, would be consistent with these findings. Another possibility is that D2R modulation of postsaccadic activity may be involved during learning of saccadic tasks, where they participate in reward-feedback based strengthening and maintenance of visuomotor associations, which would be consistent with the findings of Puig and Miller (2014). What experiments can be performed to further our understanding of the nature of saccade-related discharges that are modulated by D2R? First, it would be necessary to inactivate thalamocortical inputs from MD with experiments similar to Sommer and Wurtz (2002) and examine the impact on postsaccadic activity in PFC, while additionally manipulating D2Rs in PFC. This could confirm whether PFC D2R-sensitive postsaccadic activity is a result of corollary discharge from the superior colliculus, as is the case in FEF. Second, it is necessary to examine the effects of D2R manipulations in postsaccadic activity in PFC in tasks where the rewarded action must be maintained and integrated across trials. Finally, studying postsaccadic activity while manipulating reward contingencies of saccades, as discussed in the previous section, while manipulating D2Rs could reveal the underlying role of this activity in the variety of contexts in which the role of PFC dopamine has been examined in this review.

## Conclusion

In this review, we have described the gamut of evidence about the role that dopaminergic neuromodulatory systems play in regulating the cognitive functions of PFC in primates. While much work has been done over the past four decades on involvement of dopamine signaling in shaping PFC cognitive circuitry during delayed response task performance, new studies performed over the past decade have begun to explore the role that dopamine plays in other cognitive and executive functions in which the PFC is intimately involved, including spatial attention, maintenance and flexible switching of cognitive sets, reward and feedback representation. While consensus is emerging about the direct physiological effects of dopamine receptor subtypes in the PFC of awake behaving primates, the complexity of dopamine’s actions on PFC task activations point to heterogeneity in the functions of cortical dopamine. Further work examining potential links between what is known about dopamine’s role in reward and reinforcement learning and its modulation of “states” of activity in well-learned tasks are needed. Such studies will be of considerable significance, given the interest in dopaminergic pharmacology in the development of treatments for enhancing cognitive outcomes in patients with neuropsychiatric diseases.

## Author Contributions

SV and SE designed the experiments. SV and AJM conducted experiments and analyzed data. SV, AJM and SE wrote the manuscript.

## Conflict of Interest Statement

The authors declare that the research was conducted in the absence of any commercial or financial relationships that could be construed as a potential conflict of interest.
